# Erratum for Sze and Schloss, “Leveraging Existing 16S rRNA Gene Surveys To Identify Reproducible Biomarkers in Individuals with Colorectal Tumors”

**DOI:** 10.1128/mBio.02076-18

**Published:** 2018-10-16

**Authors:** Marc A. Sze, Patrick D. Schloss

**Affiliations:** a Department of Microbiology and Immunology, University of Michigan, Ann Arbor, Michigan, USA

## ERRATUM

Volume 9, no. 3, e00630-18, 2018, https://doi.org/10.1128/mBio.00630-18. Table 2 has two typographical errors. The number of control and carcinoma samples from the Geng et al. study should be 8 rather than 16. The analysis used the correct number of samples. The number of samples in the Sanapareddy et al. study was incorrectly placed in the carcinoma column and should have been in the adenoma column. The analysis for unmatched tissue and adenoma tissue was rerun with this correction and did not meaningfully change the results. An updated version of the analysis can be found at https://github.com/SchlossLab/Sze_CRCMetaAnalysis_mBio_2018.

