# Supplementary figures and images for: Leveraging Existing 16S rRNA Gene Surveys To Identify Reproducible Biomarkers in Individuals with Colorectal Tumors
Source: mBio. 2018 Jun 5;9(3):e00630-18. doi: 10.1128/mBio.00630-18 (PMC5989068; doi:10.1128/mBio.00630-18)

**A**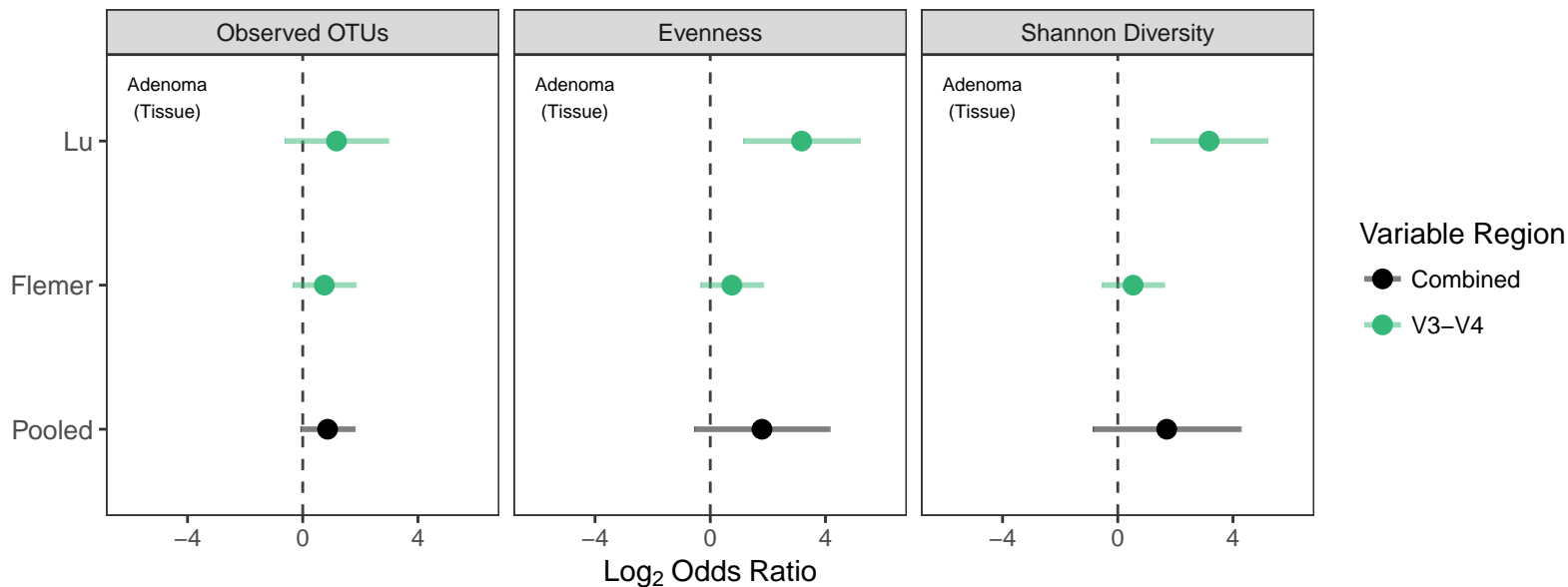**B**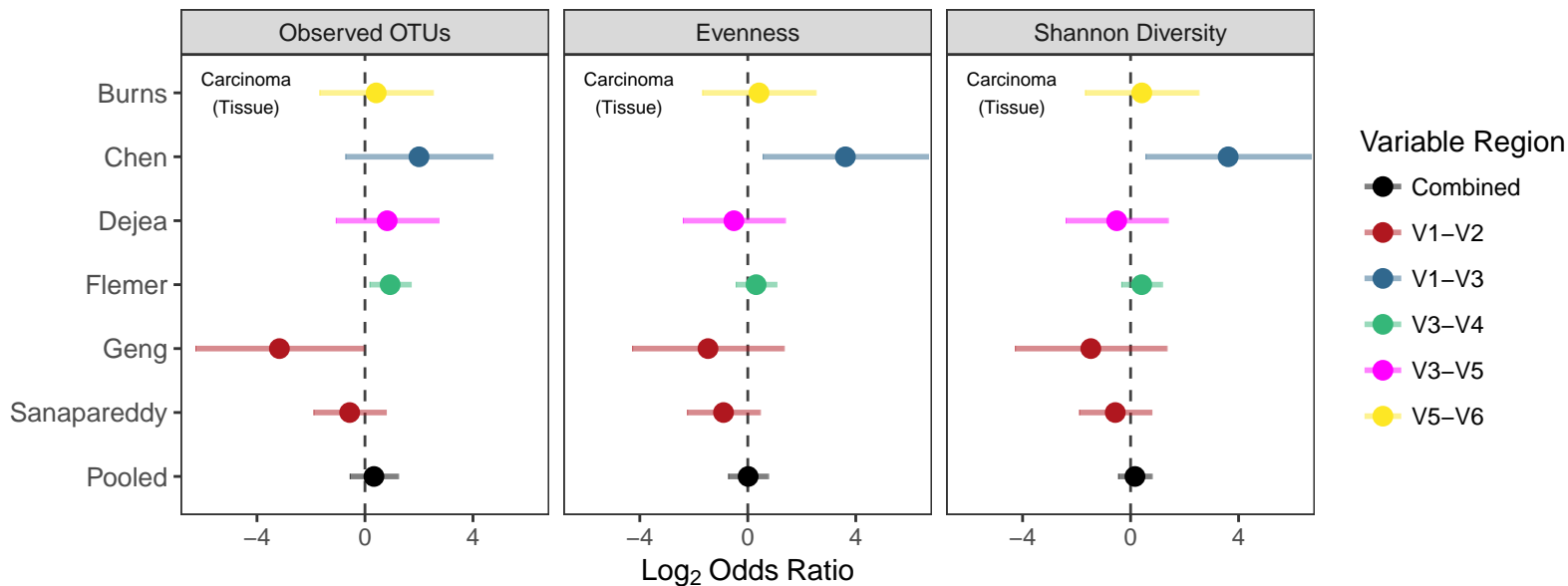

Supplement: FIG S1 [file mbo003183918sf1.pdf]

**A**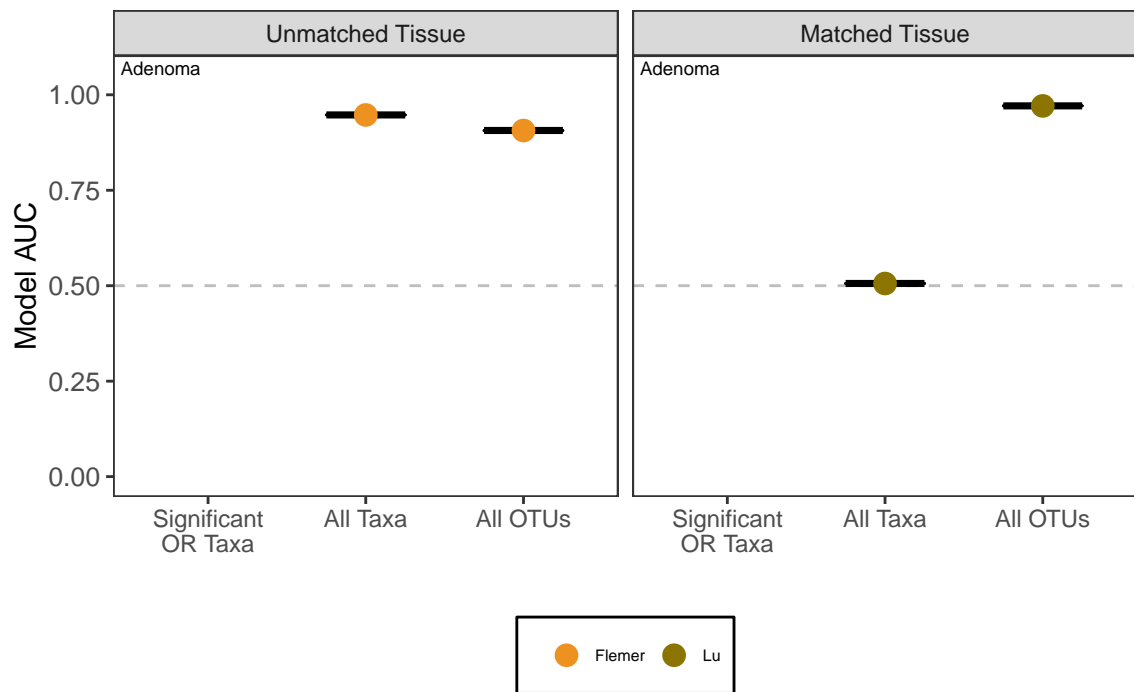**B**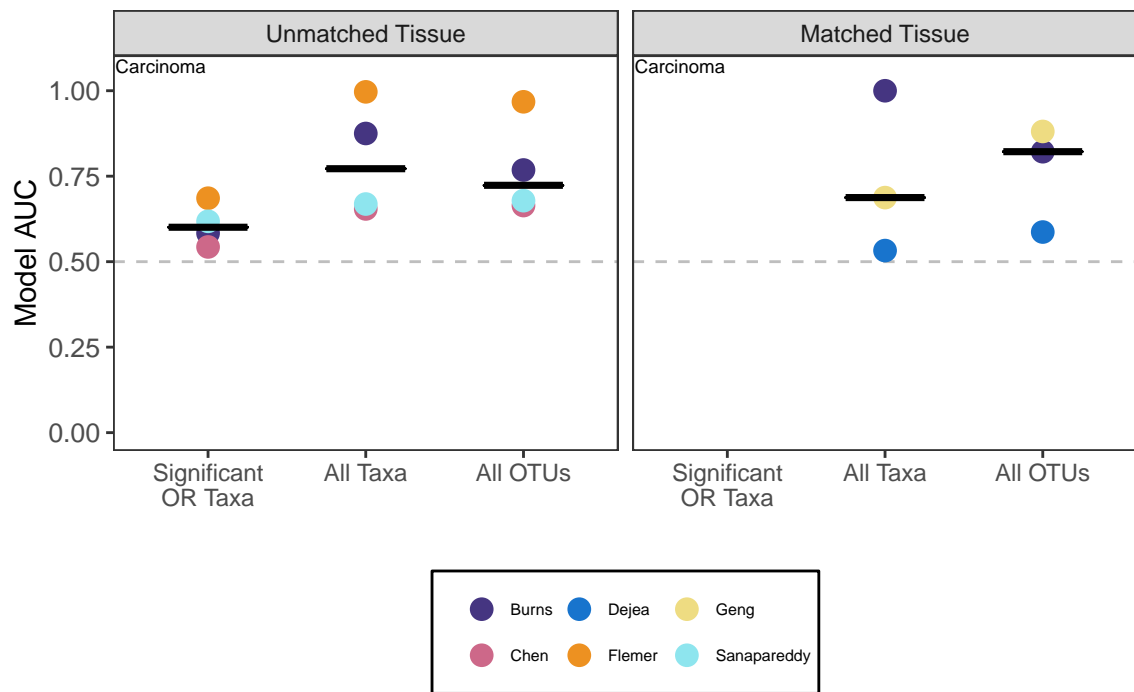

Supplement: FIG S2 [file mbo003183918sf2.pdf]

A

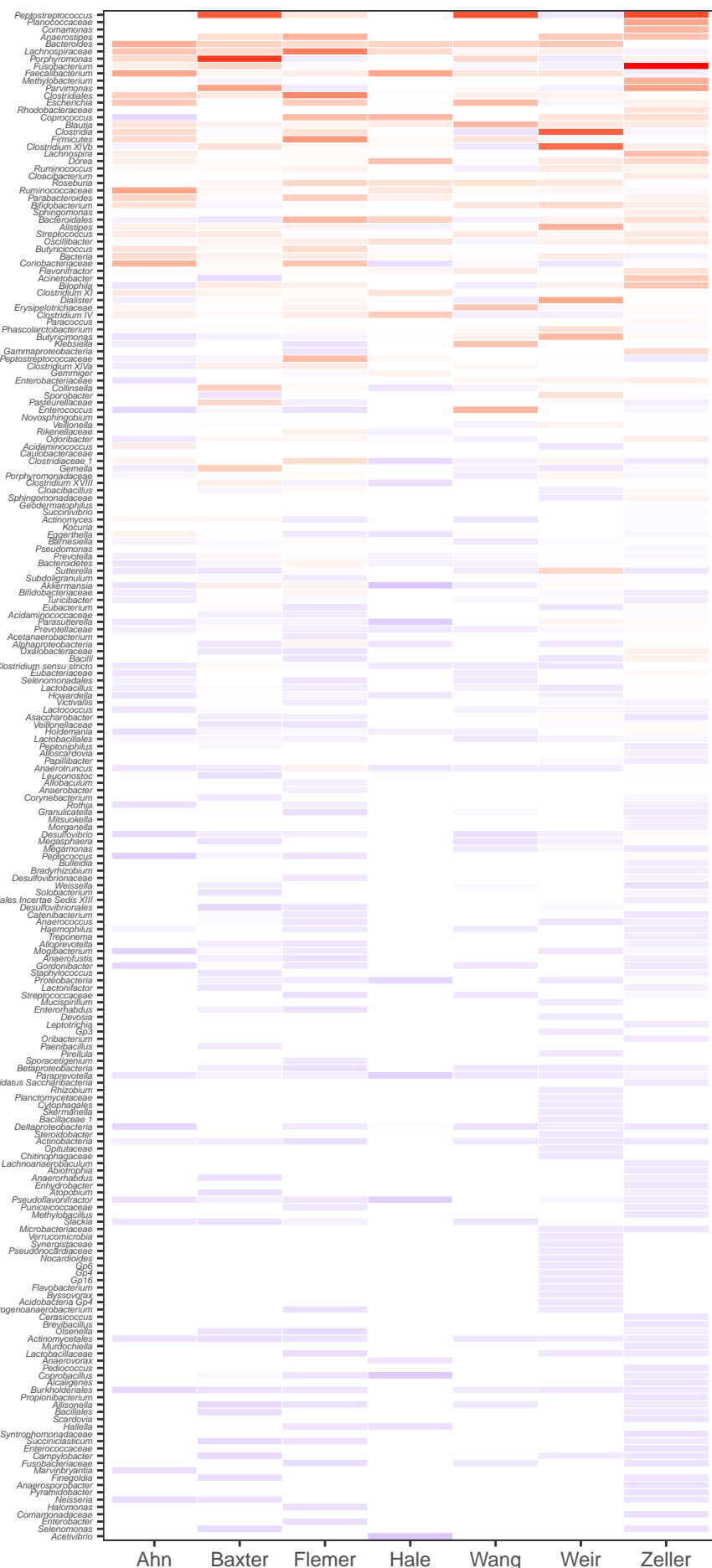

Z-Score MDA

0 2 4 6

B

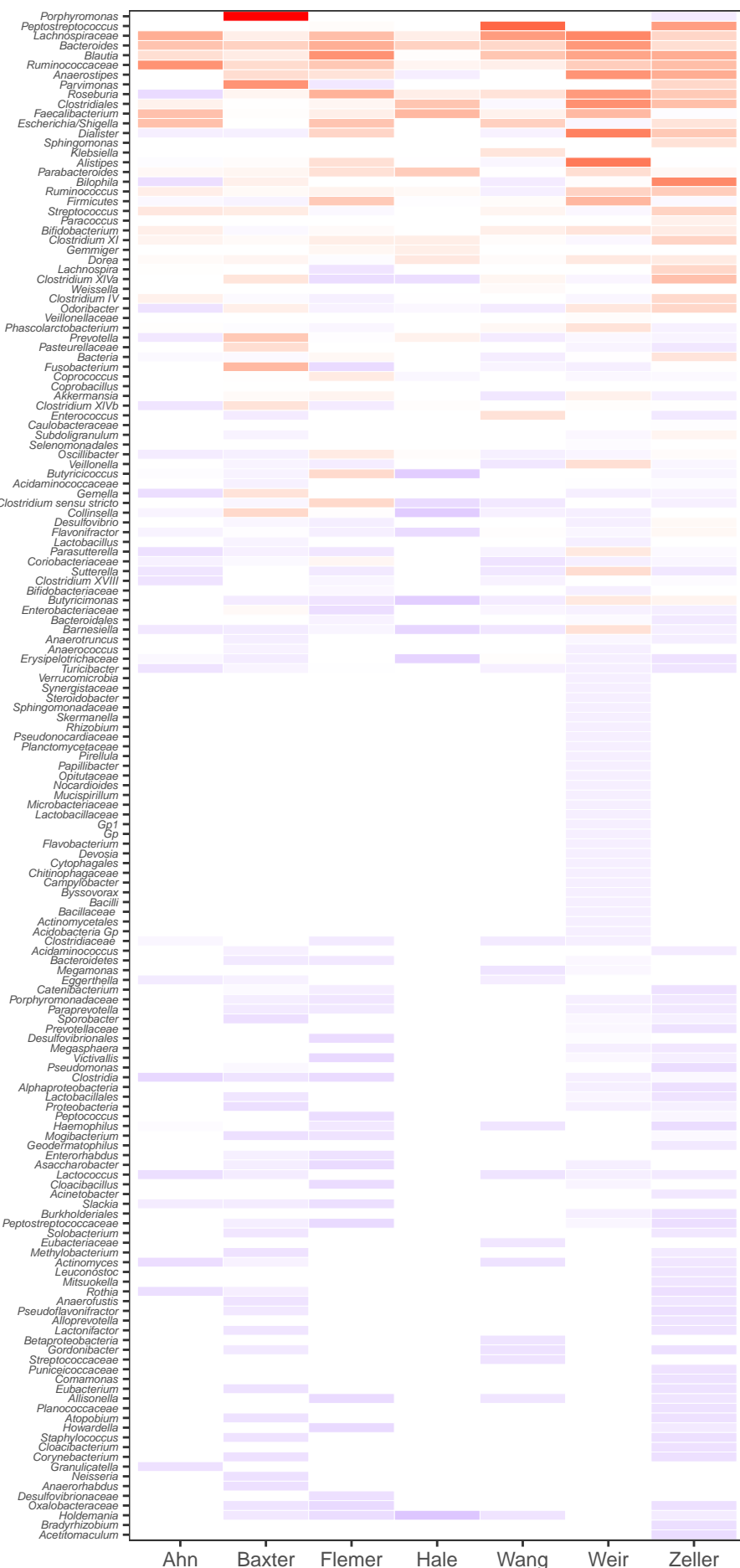

Z-Score Median MDA

0 2 4 6

Supplement: FIG S3 [file mbo003183918sf3.pdf]

**A**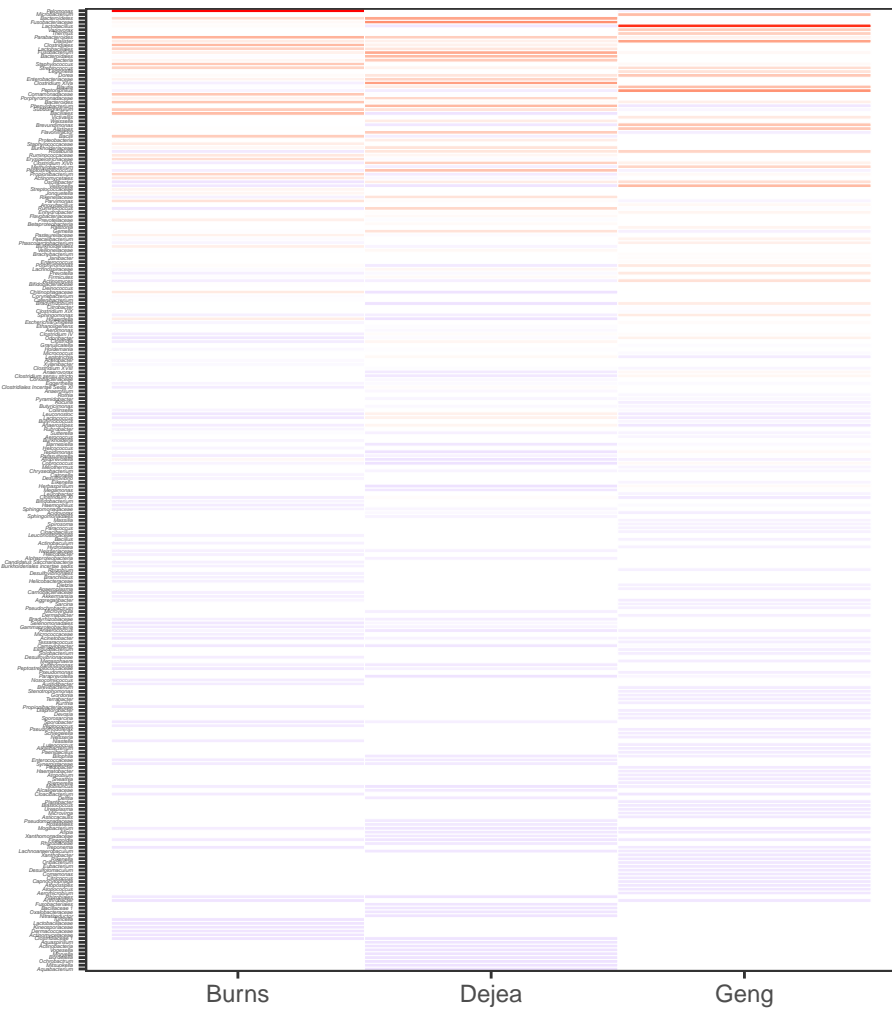

Z-Score MDA

0.0 2.5 5.0

**B**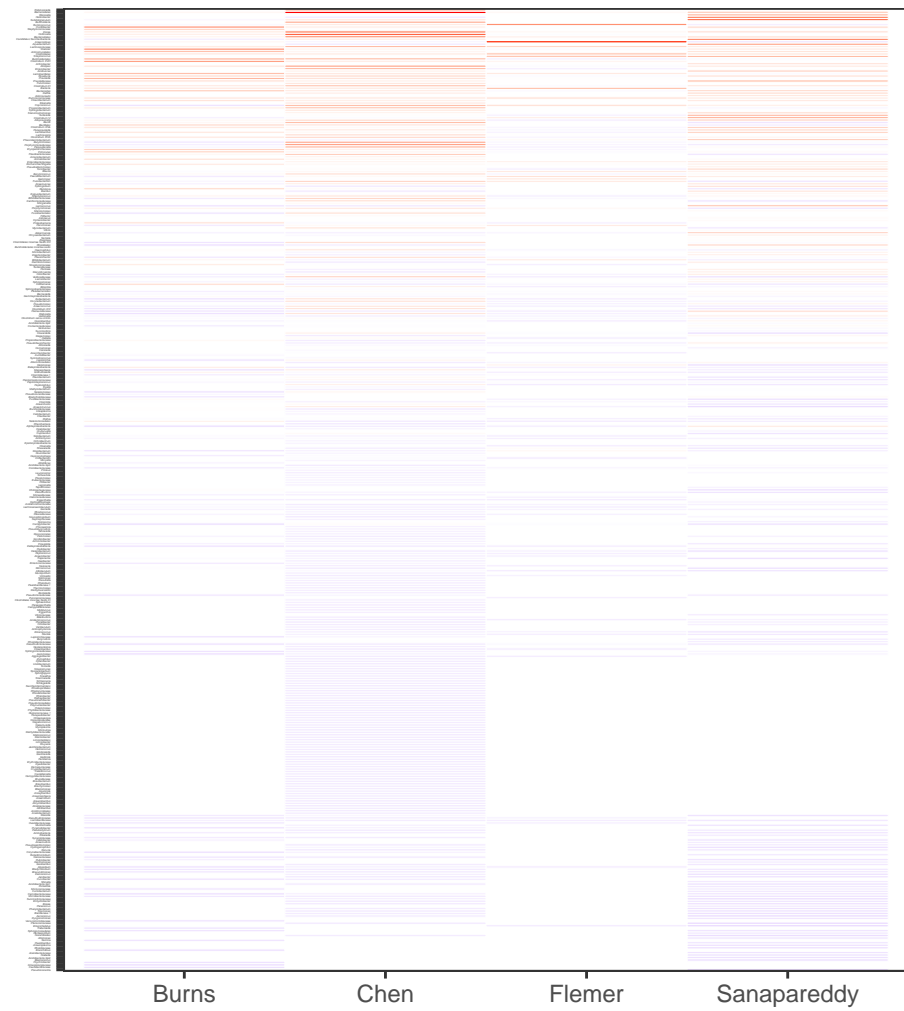

Z-Score MDA

0 2 4 6

**C**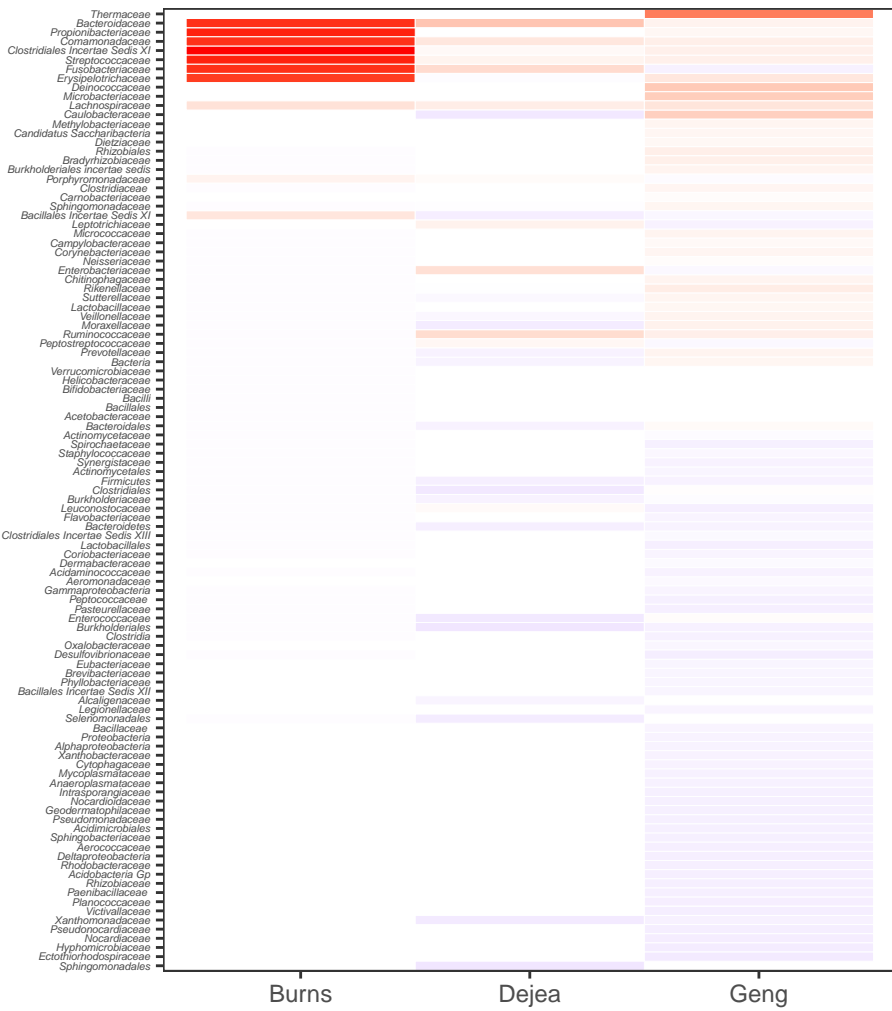

Z-Score MDA

0.0 2.5 5.0 7.5

**D**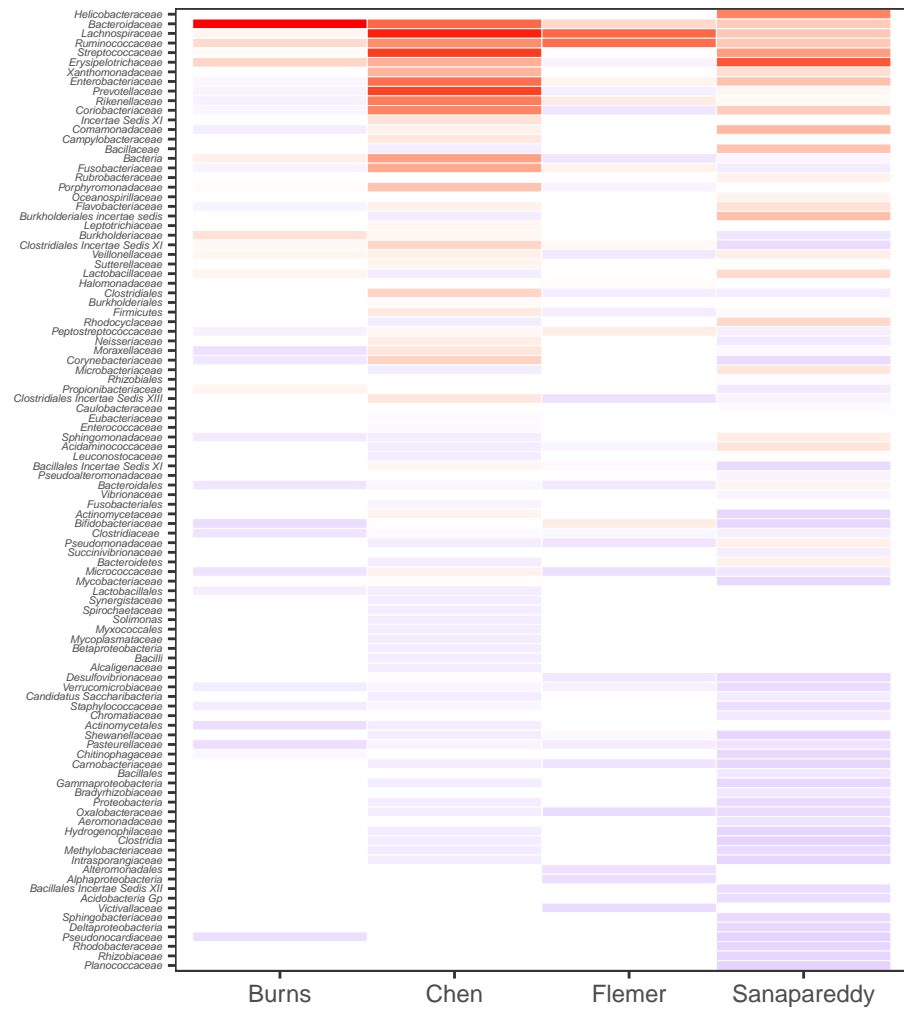

Z-Score MDA

0 1 2 3 4

Supplement: FIG S4 [file mbo003183918sf4.pdf]

**A**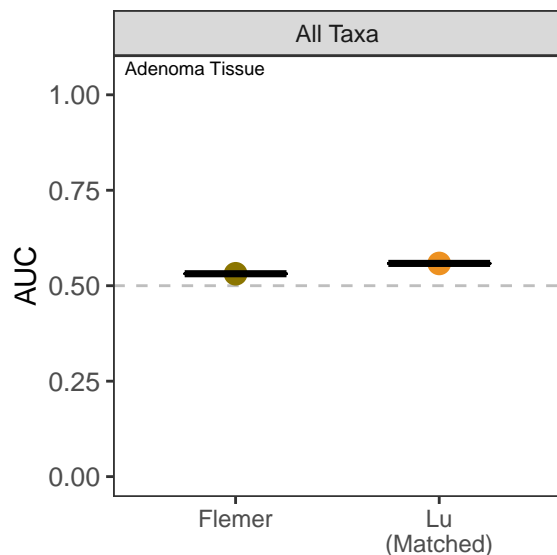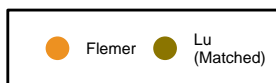**B**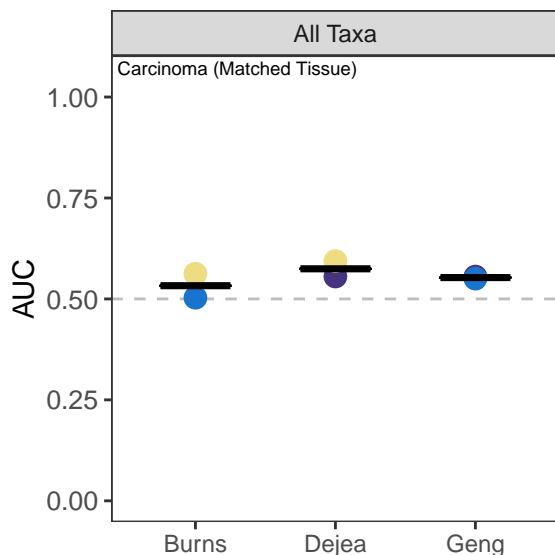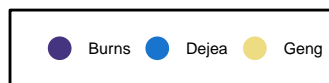**C**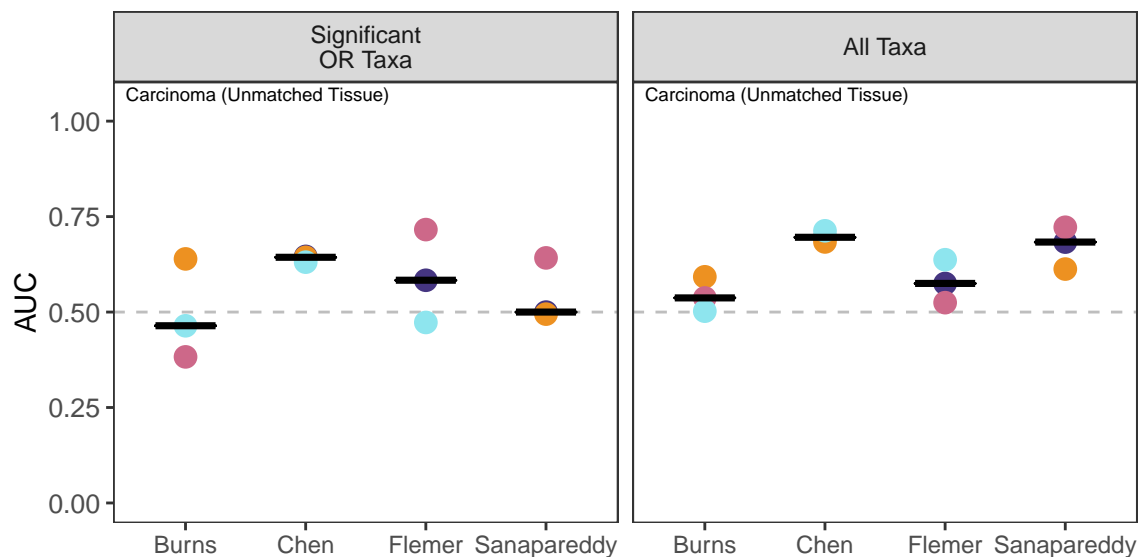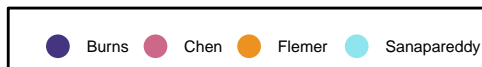

Supplement: FIG S5 [file mbo003183918sf5.pdf]
