# Supplementary material for: Leveraging Existing 16S rRNA Gene Surveys To Identify Reproducible Biomarkers in Individuals with Colorectal Tumors
Source: mBio. 2018 Jun 5;9(3):e00630-18. doi: 10.1128/mBio.00630-18 (PMC5989068; doi:10.1128/mBio.00630-18)
Supplement: TABLE S1 [file mbo003183918st1.pdf]

Table S1: Comparison of odds ratios calculated using alpha diversity community metrics associated with the presence of adenomas or carcinoma relative to those in individuals with normal colons using data collected from tissue samples.

Odds Ratio	95% CI (Lower Bound)	95% CI (Upper Bound)	P-value	Measure	Tumor	Tissue Group
1.82	0.95	3.49	0.07	OTU Richness	Adenoma	Combined
3.25	0.55	19.25	0.19	Shannon Diversity	Adenoma	Combined
3.47	0.68	17.70	0.13	Evenness	Adenoma	Combined
1.53	0.77	3.04	0.22	OTU Richness	Carcinoma	Unmatched
1.43	0.67	3.05	0.35	Shannon Diversity	Carcinoma	Unmatched
1.84	0.54	6.24	0.33	Evenness	Carcinoma	Unmatched
0.43	0.07	2.45	0.34	OTU Richness	Carcinoma	Matched
0.45	0.14	1.43	0.17	Shannon Diversity	Carcinoma	Matched
0.45	0.14	1.43	0.17	Evenness	Carcinoma	Matched
