# Supplementary material for: Leveraging Existing 16S rRNA Gene Surveys To Identify Reproducible Biomarkers in Individuals with Colorectal Tumors
Source: mBio. 2018 Jun 5;9(3):e00630-18. doi: 10.1128/mBio.00630-18 (PMC5989068; doi:10.1128/mBio.00630-18)
Supplement: TABLE S2 [file mbo003183918st2.pdf]

Table S2: Comparison of community dissimilarity between individuals with normal colons and those with adenomas and carcinomas as calculated using Bray-Curtis distance and tested using PERMANOVA.

Study	Tumor	Sample Type	R2	P-value
Brim	Adenoma	Feces	0.059	0.7562
Zeller	Adenoma	Feces	0.021	0.0096
Baxter	Adenoma	Feces	0.003	0.3788
Hale	Adenoma	Feces	0.001	0.3658
Wang	Carcinoma	Feces	0.034	0.0001
Weir	Carcinoma	Feces	0.107	0.3431
Ahn	Carcinoma	Feces	0.010	0.0033
Zeller	Carcinoma	Feces	0.028	0.0003
Baxter	Carcinoma	Feces	0.007	0.0024
Hale	Carcinoma	Feces	0.002	0.7163
Flemer	Carcinoma	Feces	0.016	0.0460
Lu	Adenoma	Tissue	0.144	0.0001
Flemer	Adenoma	Tissue	0.018	0.0001
Lu (Matched)	Adenoma	Tissue	0.569	0.1000
Sanapareddy	Carcinoma	Tissue	0.025	0.0069
Burns	Carcinoma	Tissue	0.051	0.0995
Flemer	Carcinoma	Tissue	0.029	0.0001
Chen	Carcinoma	Tissue	0.064	0.2691
Dejea (Matched)	Carcinoma	Tissue	0.048	0.2515
Geng (Matched)	Carcinoma	Tissue	0.030	0.9816
Burns (Matched)	Carcinoma	Tissue	0.168	1.0000
