# Supplementary material for: Leveraging Existing 16S rRNA Gene Surveys To Identify Reproducible Biomarkers in Individuals with Colorectal Tumors
Source: mBio. 2018 Jun 5;9(3):e00630-18. doi: 10.1128/mBio.00630-18 (PMC5989068; doi:10.1128/mBio.00630-18)
Supplement: TABLE S3 [file mbo003183918st3.pdf]

Table S3: ORs for individual taxa associated with individuals who had a normal colon or adenomas or carcinomas using data collected from stool. The listed P-values were less than 0.05 prior to using a Benjimani-Hochberg correction for multiple comparisons.

Taxon	Tumor	OR	95% CI (Lower Bound)	95% CI (Upper Bound)	P-value	BH
Clostridium_XlVb	Adenoma	1.46	1.14	1.86	2.29e-03	2.20e-01
Porphyromonas	Adenoma	1.77	1.19	2.62	4.48e-03	2.20e-01
Lachnospiraceae	Adenoma	0.71	0.56	0.91	6.40e-03	2.20e-01
Novosphingobium	Adenoma	3.33	1.27	8.72	1.41e-02	2.92e-01
Bacteroidales	Adenoma	1.35	1.06	1.72	1.63e-02	2.92e-01
Clostridium_XI	Adenoma	0.75	0.59	0.95	1.92e-02	2.92e-01
Clostridiaceae_1	Adenoma	0.71	0.54	0.95	1.99e-02	2.92e-01
Lactococcus	Adenoma	0.68	0.47	0.97	3.56e-02	4.59e-01
Porphyromonas	Carcinoma	3.20	2.26	4.54	6.73e-11	5.59e-09
Peptostreptococcus	Carcinoma	7.11	3.84	13.17	4.60e-10	1.91e-08
Parvimonas	Carcinoma	3.07	2.11	4.46	3.80e-09	1.05e-07
Fusobacterium	Carcinoma	2.74	1.95	3.85	5.54e-09	1.15e-07
Escherichia.Shigella	Carcinoma	2.15	1.57	2.95	2.20e-06	3.65e-05
Enterobacteriaceae	Carcinoma	1.79	1.33	2.41	1.30e-04	1.80e-03
Ruminococcus	Carcinoma	0.63	0.48	0.83	1.19e-03	1.41e-02
Clostridium_XI	Carcinoma	0.65	0.49	0.86	2.94e-03	3.05e-02
Roseburia	Carcinoma	0.60	0.41	0.88	8.98e-03	8.28e-02
Clostridium_XlVb	Carcinoma	1.45	1.09	1.94	1.17e-02	9.72e-02
Clostridiaceae_1	Carcinoma	0.67	0.48	0.93	1.53e-02	1.15e-01
Campylobacter	Carcinoma	1.76	1.10	2.82	1.88e-02	1.30e-01
Anaerococcus	Carcinoma	2.50	1.14	5.47	2.22e-02	1.42e-01
Desulfovibrio	Carcinoma	1.45	1.05	2.00	2.54e-02	1.50e-01
Veillonellaceae	Carcinoma	1.53	1.04	2.26	3.28e-02	1.79e-01
Lachnospiraceae	Carcinoma	0.69	0.49	0.97	3.44e-02	1.79e-01
