# Supplementary material for: Leveraging Existing 16S rRNA Gene Surveys To Identify Reproducible Biomarkers in Individuals with Colorectal Tumors
Source: mBio. 2018 Jun 5;9(3):e00630-18. doi: 10.1128/mBio.00630-18 (PMC5989068; doi:10.1128/mBio.00630-18)
Supplement: TABLE S4 [file mbo003183918st4.pdf]

Table S4: ORs for individual taxa associated with individuals who had a normal colon or adenomas or carcinomas using data collected from tissue samples. The listed P-values were less than 0.05 prior to using a Benjimani-Hochberg correction for multiple comparisons.

Taxon	Tumor	Tissue Group	OR	95% CI (Lower Bound)	95% CI (Upper Bound)	P-value	BH
Lachnospiraceae	Adenoma	Combined	0.27	0.14	0.56	3.23e-04	7.17e-02
Pseudomonas	Adenoma	Combined	3.73	1.73	8.05	8.13e-04	7.17e-02
Howardella	Adenoma	Combined	3.69	1.72	7.95	8.30e-04	7.17e-02
Rothia	Adenoma	Combined	2.87	1.48	5.58	1.82e-03	1.18e-01
Blautia	Adenoma	Combined	0.39	0.20	0.77	6.96e-03	3.61e-01
Enterobacter	Adenoma	Combined	3.42	1.28	9.12	1.42e-02	4.65e-01
Puniceicoccaceae	Adenoma	Combined	2.94	1.22	7.10	1.61e-02	4.65e-01
Erysipelotrichaceae	Adenoma	Combined	2.27	1.16	4.42	1.62e-02	4.65e-01
Streptococcus	Adenoma	Combined	2.27	1.16	4.42	1.62e-02	4.65e-01
Lactococcus	Adenoma	Combined	3.24	1.17	9.00	2.37e-02	6.15e-01
Micrococcaceae	Adenoma	Combined	3.91	1.17	13.06	2.65e-02	6.24e-01
Shewanella	Adenoma	Combined	2.05	1.05	3.98	3.46e-02	6.66e-01
Phascolarctobacterium	Adenoma	Combined	2.03	1.05	3.93	3.57e-02	6.66e-01
Achromobacter	Adenoma	Combined	7.10	1.14	44.44	3.61e-02	6.66e-01
Anaerostipes	Adenoma	Combined	0.10	0.01	0.89	3.96e-02	6.66e-01
Neisseria	Adenoma	Combined	2.09	1.00	4.35	4.88e-02	6.66e-01
Fusobacterium	Adenoma	Combined	2.85	1.00	8.12	4.94e-02	6.66e-01
Dorea	Carcinoma	Unmatched	0.35	0.22	0.55	3.96e-06	4.40e-04
Weissella	Carcinoma	Unmatched	5.15	2.02	13.14	5.96e-04	2.88e-02
Blautia	Carcinoma	Unmatched	0.47	0.30	0.73	7.79e-04	2.88e-02
Campylobacter	Carcinoma	Unmatched	2.13	1.23	3.67	6.57e-03	1.80e-01
Leptotrichia	Carcinoma	Unmatched	2.71	1.30	5.67	8.10e-03	1.80e-01
Parvimonas	Carcinoma	Unmatched	1.94	1.17	3.21	1.05e-02	1.95e-01
Clostridiaceae_1	Carcinoma	Unmatched	1.99	1.15	3.45	1.40e-02	2.15e-01
Flavobacteriaceae	Carcinoma	Unmatched	2.34	1.17	4.69	1.64e-02	2.15e-01
Ruminococcus2	Carcinoma	Unmatched	0.28	0.10	0.81	1.81e-02	2.15e-01
Clostridium_XlVb	Carcinoma	Unmatched	0.60	0.39	0.92	1.95e-02	2.15e-01
Corynebacterium	Carcinoma	Unmatched	0.52	0.29	0.92	2.41e-02	2.15e-01
Finegoldia	Carcinoma	Unmatched	0.43	0.21	0.90	2.45e-02	2.15e-01
Lachnospiraceae	Carcinoma	Unmatched	0.43	0.21	0.90	2.52e-02	2.15e-01
Bacteroides	Carcinoma	Unmatched	0.48	0.26	0.92	2.71e-02	2.15e-01
Clostridium_sensu_stricto	Carcinoma	Unmatched	1.89	1.04	3.43	3.79e-02	2.80e-01
Barnesiella	Carcinoma	Unmatched	0.64	0.41	0.98	4.16e-02	2.89e-01
Fusobacterium	Carcinoma	Matched	3.98	1.19	13.24	2.45e-02	9.26e-01
Campylobacter	Carcinoma	Matched	7.80	1.20	50.88	3.18e-02	9.26e-01
